# Effects of moisture and fermentation length on the quality and digestibility of fermented concentrate using tamanu kernel cake as the main protein source through an *in vitro* study

**DOI:** 10.5455/javar.2025.l910

**Published:** 2025-05-07

**Authors:** Dimas Hand Vidya Paradhipta, Chusnul Hanim, Ali Agus, Budi Leksono, Aziz Umroni, Sinta Maharani, Arrynda Rachma Dyasti Wardani, Zazin Mukmila

**Affiliations:** 1 Department of Animal Feed and Nutrition, Faculty of Animal Science, Universitas Gadjah Mada, Yogyakarta, Indonesia; 2 Research Center for Applied Botany, National Research and Innovation Agency (BRIN), Cibinong, Indonesia; 3 Research Center for Applied Zoology, National Research and Innovation Agency (BRIN), Cibinong, Indonesia

**Keywords:** Fermentation quality, fermented concentrate, fermentation length, moisture level, ruminal digestibility, tamanu kernel cake

## Abstract

**Objective:**

The present study aimed to evaluate the effects of moisture and fermentation length on the chemical compositions, fermentation characteristics, feed-out phase, and ruminal digestibility of fermented concentrate using tamanu kernel cake (TKC) as the main protein source.

**Materials and Methods:**

The concentrate was formulated to contain 16.5% crude protein (CP) and 35% neutral detergent fiber consisting of 40% TKC, 7.60% soybean meal, 25.0% wheat pollard, 26.4% dried cassava, and 1% molasses. Those ingredients were mixed and fermented anaerobically at 5 kg into a vacuumed plastic bag with different additional sterile distilled water to reach moisture levels at 25% (MO25), 35% (MO35), and 45% (MO45). Each moisture level was incubated with different fermentation lengths consisting of 2, 7, 14, 21, and 42 days in quadruplicate. After fermentation, each silo was sub-sampled for laboratory analyses.

**Results:**

MO25 and MO35 led to higher CP with lower acid detergent fiber than MO45. In the fermentation, MO35 and MO45 generated higher (*p* < 0.05) lactate than MO25. An extended fermentation length linearly dropped (*p* < 0.05) dry matter, CP, and ether extract, but gradually increased (*p* < 0.05) ammonia-N, lactate, acetate, and the counts of lactic acid bacteria, yeast, and bacilli. After 42 days, MO25 and MO35 initiated higher (*p* < 0.05) aerobic stability. The digestibility and total volatile fatty acid (VFA) in the rumen increased (*p* < 0.05) over 2 days. However, prolonged fermentation length linearly decreased (*p* < 0.05) total VFA and methane emission without affecting rumen pH, ammonia-N, and each VFA profile.

**Conclusion:**

The application of MO35 was found to reduce nutrient loss and improve aerobic stability comparable to MO25 while achieving fermentation quality similar to MO45, and short-term fermentation, such as 2 days, could improve ruminal digestibility.

## Introduction

In Indonesia, ruminant feed is mainly prepared with by-products commonly containing high concentrations of neutral detergent fiber (NDF), including ingredients used in concentrate. Ruminant farmers commonly use fermentation techniques to enhance concentrate quality, as it mainly consists of agricultural and plantation by-products as ingredients. Despite containing high protein or energy, high NDF content in the concentrate limits nutrient palatability and digestibility. Reports indicate that fermenting concentrate with complex microbes, such as probiotics, enhances animal performance [1–3]. This improvement tends to be due to fermented concentrate serving as a re-culture medium for the growth of probiotics. In addition, fibrinolytic microbes from the inoculant can help degrade NDF concentration in the diet, thereby increasing palatability and digestibility [4]. As for production, some concentrated ingredients have an undesirable odor caused by residual plant secondary metabolites. The fermentation process also helps to improve physical condition [5], especially undesirable odor, by reducing the concentration of plant secondary metabolites [6], which could enhance palatability for animals.

The production of fermented concentrate has recently expanded beyond the farmer level to the industrial scale. During mass production, the quality of fermentation for animal feed varies due to several factors, such as differences in energy level, buffering capacity, moisture content, and length of fermentation [7–10]. In the field, the moisture level and the length of fermenting concentrate are applied in many variations. Generally, concentrate fermentation is conducted in low moisture content, lower than 45%, while the obtained concentrate can be stored for 2 months, and these methods help to reduce nutrient loss during storage. Higher moisture levels can promote the growth of desirable microbes, including lactic acid bacteria (LAB), as well as fibrinolytic and proteolytic microbes [11–15], but increase nutrient loss and reduce aerobic stability [10,16,17]. The length of fermentation is related to the growth phase of LAB, whose growth rate depends on diet chemical composition [8–11,18]. Studies on the optimal moisture level and length of fermentation for the concentrate have limited information, specifically regarding high crude protein (CP) content ≥ 16%. High protein content tends to increase ammonia-N production during fermentation, which affects the buffering capacity of fermented concentrate.

Tamanu kernel cake (TKC) is a promising new alternative feedstuff with high protein content for animals, particularly ruminants [19]. Furthermore, it is a by-product of tamanu oil used for biodiesel, which recently gained popularity in Indonesia. Tamanu trees (Calophyllum inophyllum) are highly adaptable to tropical climates [20], and as a by-product, TKC production increases with oil demand [19]. Based on the 135-hectare tamanu plantation in Purworejo, which serves as a coastal windbreak, and considering a dry seed production potential of 1.87 tons per hectare [20], with half of this yield consisting of TKC, the estimated annual TKC production in Purworejo could reach approximately 126.2 tons. TKC contains high NDF concentration and remains as plant secondary metabolites affecting odor, thereby reducing palatability and digestibility [19]. The application of this feedstuff in the form of fermented concentrate is an option to improve the palatability of ruminants, leading to its being used as the main protein source to formulate high-protein concentrate. The improvement of the nutritive value of concentrate-based TKC can occur by fermentation. However, high protein content in TKC might be a limiting factor for the successful fermentation of concentrate. The outcomes of moisture and fermentation length on the concentrate need to be evaluated to provide a recommendation for industry-scale product development, especially for high-protein concentrate that could have limitations in buffering capacity during ensiling. Therefore, the present study had a purpose to investigate the effects of differences in moisture level and fermentation length on the chemical compositions, fermentation characteristics, feed-out phase, and ruminal digestibility of high-protein fermented concentrate using TKC as the main ingredient. In addition, the objective of the present study was to find a proper moisture and fermentation length for fermenting high-protein fermented concentrate based on TKC.

## Material and Method

### Ethical approval

For the *in vitro* study, the animal care of cannulated cattle was in agreement with and approved by the Legal Ethical Committee, Integrated Laboratory for Research and Testing, Universitas Gadjah Mada (No. 00056/04/LPPT/ XII/2023; date: 4 December 2023).

### Production of fermented concentrate

The concentrate in this study was formulated to contain 16.5% CP and 35% NDF, with TKC as the main ingredient protein source. TKC was collected from the tamanu oil industry located in the Purworejo area, Central Java, Indonesia, and then analyzed for chemical composition according to AOAC [21]. Other ingredients consisted of dried cassava, wheat pollard, soybean meal, and molasses, with the respective proportions in the ration presented in Table 1. All ingredients were mixed, and the moisture content of the concentrate was around 15.2%. The concentrate was fermented using the addition of commercial inoculant at different moisture levels consisting of 25% (MO25), 35% (MO35), and 45% (MO45), while sterile distilled water was added to achieve the target moisture level. The commercial inoculant contained a mixture of *Lactobacillus* sp., *Saccharomyces cerevisiae*, and *Bacillus subtilis*, which was applied at 1 × 10^5^ colony forming unit (cfu)/ml into the fermented concentrate. Fermentation was conducted in 5 kg using a mini-silo in anaerobic conditions, and each moisture level was fermented for different durations, consisting of 2, 7, 14, 21, and 42 days. Both treatments of moisture levels and fermentation length used four silos as replication. On the assigned day, each fermented concentrate was compressed at 200 gm for analyses of chemical composition according to proximate methods. Other silage was compressed to approximately 20 gm for analyses of fermentation characteristics and microbial counts. On day 42, a total of 1 kg of fermented concentrate was collected from each mini silo for analysis of aerobic stability. The sample with the recommended moisture level was incubated in a rumen buffer to investigate the effects of fermentation length on digestibility.

**Table 1.** Chemical composition of tamanu kernel cake in the present study (%, DM).

Item	
Dry matter	90.0 ± 0.12
Organic matter	91.9 ± 0.14
Crude protein	20.2 ± 0.14
Ether extract	15.3± 0.29
Neutral detergent fiber	38.0 ± 0.17
Acid detergent fiber	15.3 ± 0.17

## Chemical composition analyses

A total of 10 gm of concentrate, both before and after anaerobic fermentation, was placed into an oven at 105°C for 24 h to measure dry matter (DM) concentration (method number 934.01; AOAC [21]). Around 190 gm of sub-sampled concentrate was prepared by drying at a medium temperature, around 60°C, for 48 h. After, the size of the sample was homogenized and reduced through a Wiley mill with a 1 mm screen. The ground sample could be used for other proximate analyses such as crude ash (CA), CP, ether extract (EE), neutral detergent fiber (NDF), and acid detergent fiber (ADF). A total of 1 gm of the sample was burned in a muffle furnace at 550°C for 5 h to determine the concentration of CA (method number 942.05; AOAC [21]). In addition, the measurement of CP was conducted based on the procedure of Kjeldahl, which consisted of destruction, distillation, and titration (method number 984.13; AOAC [21]). The Soxhlet method was used to determine the concentration of EE (method number 920.39; AOAC [21]). The fiber fractions consisting of NDF and ADF were determined according to the procedure of Van Soest (method numbers 2002.04 and 973.13, respectively; AOAC [21]) using ANKOM Fiber Digestion (ANKOM 200 Fiber Analyzer, USA). The chemical composition of TKC was also investigated following similar procedures of AOAC [21].

## Fermentation characteristics analysis

A total of 180 ml of ultrapure distilled water was mixed with 20 gm concentrate using a blender for 30 sec to conduct the extraction. The extraction was used to analyze pH, ammonia-N, and organic acid profiles. A pH meter (Mettler Toledo LE438, USA) was used to measure the pH value of all fermented concentrates. The principle of colorimetry, according to Chaney and Marbach [22], was applied to measure the concentration of ammonia-N in all fermented concentrates. The determination of lactate was evaluated based on the method of Barker and Summerson [23] using a UV-VIS spectrophotometer. The extraction was centrifuged at 12,000 rpm to separate the supernatant and residue. The supernatant was collected and prepared for analyses of VFA profiles using gas chromatography (GC-8A, Shimadzu Corp., Japan) following the procedure of Filipek and Dvorak [24]. The measurement of VFA profiles in the present study consisted of acetate, propionate, and butyrate. The concentrations of fermentation variables, consisting of ammonia-N, lactate, and each VFA profile, were presented as % of DM.

## Microbiological count analysis

The extraction of fermented concentrate was used to analyze microbial counts. The dilution series of extraction was applied until 10^-7^. Generally, dilution series of 10^-3^ to 10^-7^ were injected into growth media. The LAB, yeast, mold, bacillus, and clostridia were enumerated in different solid mediums. The de Man, Rogosa, and Sharpe (MRS; Sigma Aldrich, USA) agar was used to cultivate LAB. Yeast and mold could be cultivated into Potato Dextrose Agar (PDA; Sigma Aldrich, USA) according to a previous study [19]. Luria–Bertani (LB) agar (Sigma Aldrich, USA). *Escherichia coli* and *Salmonella* were cultivated on violet red bile (VRB) agar (Sigma Aldrich, USA) and *Salmonella*-Shigella (SS) agar (Sigma Aldrich, USA), respectively. For counting LAB, all plates of MRS agar were stored in an anaerobic incubator [17,25]. For counting aerobic microbes, all plates of PDA, LB, VRB, and SS agar were stored in an aerobic incubator [3]. The temperature of incubators, both anaerobic and aerobic, was set at 30°C, and all plates were stored for 48 h [3,17,25]. The cfu per gram was used to express the count of each microbe, and then it was calculated to log10.

## Aerobic stability

A total of 1 kg sample was transferred into a mini silo measuring 5 L under aerobic conditions. The temperature of the sample was recorded every 3 h. The observation of temperature would be stopped if the temperature in the sample was 2°C higher than the ambient temperature. Aerobic stability was calculated based on the time (h) required for the silage temperature to rise by 2°C above ambient [25,18].

## Ruminal incubation

Samples with selected moisture levels from different fermentation lengths, consisting of 0, 2, 7, 14, 21, and 42 days, were incubated in rumen buffer. For animal donors, two cannulated cattle of the Bali breed (*Bos indicus*) were used in the present study as a source of rumen fluid. For the information, the average weight of two Bali cattle was 310 kg. Both cattle were fed a diet containing CP at 12% and ME at 10 kcal/kg. The diet consisted of roughage (Napier grass) and commercial concentrate (mixed grains), which was applied at a 7:3 ratio, ensuring isonitrogenous and isoenergetic conditions. Before morning feeding, a total of 2 L of rumen fluid was collected from donor cattle. A loosely woven gauze in two layers was used to filter a collected rumen fluid, which was mixed with an anaerobic culture medium at a 1:2 ratio as defined by Adesogan et al. [26], and this mixture was called rumen buffer. Each sample was weighed at 500 mg and then placed into a 100 ml incubation bottle, and 40 ml of rumen buffer was poured out. CO₂ was applied in all incubation bottles through gassing for 30 sec to reach anaerobic conditions. After the incubation bottle was closed tightly. Each treatment was applied in quadruplicate using 4 bottles along with four blanks. The incubation was conducted at 39°C using an incubator, and then *in vitro* incubation was conducted in two batches as a replication of the period.

## Ruminal fermentation analyses

The glass wool was placed into the Gooch crucibles to screen the remaining sample and the rumen buffer after incubation. Post-gas collection, all bottles were opened and filtered with Gooch crucibles, then the filtered sample was used to calculate *in vitro* dry matter digestibility (IVDMD) and *in vitro* organic matter digestibility (IVOMD) according to Paradhipta et al. [19]. Filtered rumen buffer was used to determine pH using a pH meter (Mettler Toledo LE438, USA), ammonia-N using the method of Chaney and Marbach [22], and VFA using gas chromatography (GC-8A, Shimadzu Corp., Japan) following the procedure of Filipek and Drovak [24]. Before, the filtered rumen buffer was centrifuged at 12,000 rpm to separate the supernatant and the remaining feed particles.

## Methane emission analyses

The emission of methane (CH_4_) was determined by collecting 10 ml of ruminal fermentation gas from each bottle using a disposable syringe after 48 h of incubation and transferring it into a vacuum tube for storage before analysis. The concentration of CH₄ was measured using gas chromatography (Shimadzu GC-2010) supported with additional instruments, such as a thermal conductivity detector (TCD), RT-OBond 30 μm capillary column, 10 μm DF, and 0.32 nm ID. The flow rate of helium as the gas carrier was applied at 1.5 ml/min, and the temperature condition was set at 50°C with a split ratio of 20. The temperature of the TCD was operated at 200°C with a current of 40 mA, while the temperature of the injector was set at 100°C. The measurement of CH₄ was presented as parts per million (ppm).

## Statistical analysis

Collected data of chemical composition, fermentation characteristics, and microbial counts were analyzed based on a factorial design with a 3 (moisture level; MO25 *vs.* MO35 *vs*. MO45) × 5 (fermentation length; 2 days *vs*. 7 days *vs.* 14 days *vs*. 21 days *vs*. 42 days) arrangement using PROC MIX of SAS [27]. The model generated was *Yijk = μ + αi + βj +* (*αβ) ij + eijk*, where *Yijk* = response variable, *μ* = overall mean, *αi* = the effect of moisture level, *βj* = the effect of fermentation length, (*αβ)ij =* the interaction effect of moisture level and fermentation length, and *eijk* = error term. The data on aerobic stability and ruminal *in vitro* digestibility were analyzed based on a completely randomized design (one-way ANOVA) by PROC ANOVA of SAS [27]. The model for this was *Yij = μ + Ti + eij,* where *Yij* = response variable, *μ* = overall mean, *Ti* = the effect of moisture level/fermentation length *i*, and *eij* = error term. Linear and quadratic contrasts were measured by PROC GLM of SAS [27] using a model of polynomial contrast based on orthogonal coefficients to evaluate the effects of fermentation length. Before, the coefficients of orthogonality were calculated to regulate an uneven spacing of fermentation length as a treatment. In post-hoc analysis, the Tukey test was applied to decide the significant difference (*p* ≥ 0.05) between treatments.

## Result

### Chemical compositions

The total chemical compositions of the entire TKC used are appropriately presented in Table 2. The TKC in the present study contained 90.0% DM, 91.9% OM, 20.2% CP, 15.3% EE, 38.0% NDF, and 15.3% ADF. As an alternative protein source, TKC was mixed with other ingredients to make a fermented concentrate with different moisture levels. Before fermentation, DM contents of MO25, MO35, and MO45 were 75%, 65.0%, and 55.1%, respectively (Table 3). Additionally, the average concentrations of OM, CP, EE, NDF, and ADF in the concentrate across all moisture levels were 89.5%, 16.5%, 7.13%, 35.2%, and 19.5%, respectively.

**Table 2.** Feed ingredients and formulation of concentrate in the present study.

Feedstuffs	Proportion (%, DM)
Tamanu kernel cake	40.0
Dried cassava	26.4
Wheat pollard	25.0
Soybean meal	7.60
Molasses	1.00
Total	100
DM, dry matter.	

**Table 3.** Chemical compositions of fermented concentrate using tamanu cake as protein source before ensiling (%, DM).

Item	Moisture level^1^		
	MO25	MO35	MO45
Dry matter	75.0 ± 0.13	65.0 ± 0.04	55.1 ± 0.04
Organic matter	89.4 ± 0.27	89.4 ± 0.31	89.5 ± 0.06
Crude protein	16.5 ± 0.06	16.5 ± 0.02	16.6 ± 0.06
Ether extract	7.10 ± 0.05	7.15 ± 0.07	7.12 ± 0.05
Neutral detergent fiber	35.0 ± 0.12	35.2 ± 0.59	35.3 ± 0.38
Acid detergent fiber	19.5 ± 0.09	19.5 ± 0.11	19.5 ± 0.18

^1^MO25, fermented concentrate with moisture level at 25%; MO35, fermented concentrate with moisture level at 35%; MO45, fermented concentrate with moisture level at 45%.

**Table 4.** Effects of moisture levels, content, and fermentation lengths on chemical compositions of fermented concentrate using tamanu cake as protein source.

Fermentation length	Chemical compositions^1^, % DM					
	DM	OM	CP	EE	NDF	ADF
MO25^2^						
2 days	74.3^a^	88.4	16.2^a^	7.12^a^	35.0^b^	19.8^ab^
7 days	74.5^a^	87.7	15.8^a^	6.37^ab^	36.3^ab^	19.8^ab^
14 days	74.0^a^	87.7	16.0^a^	6.31^ab^	36.4^ab^	20.4^ab^
21 days	73.7^a^	88.1	16.0^a^	6.14^ab^	37.7^ab^	21.5^ab^
42 days	72.8^a^	87.8	15.2^a^	6.17^ab^	39.5^a^	21.0^ab^
MO35						
2 days	64.1^b^	87.8	16.7^a^	7.09^a^	35.2^b^	18.5^b^
7 days	63.7^b^	87.9	16.4^a^	6.88^ab^	37.0^ab^	19.9^ab^
14 days	62.8^b^	88.4	16.0^a^	6.57^ab^	38.1^ab^	20.6^ab^
21 days	62.9^b^	88.5	15.7^a^	6.78^ab^	39.5^a^	21.4^ab^
42 days	60.4^c^	87.5	14.8^ab^	5.48^b^	40.0^a^	21.9^ab^
MO45						
2 days	54.1^d^	88.9	16.1^a^	7.11^a^	34.8^b^	20.9^ab^
7 days	53.2^d^	89.5	15.8^a^	6.77^ab^	36.0^b^	20.8^ab^
14 days	52.6^d^	88.5	14.7^ab^	6.46^ab^	36.7^ab^	21.4^ab^
21 days	52.4^d^	89.3	14.1^b^	6.06^b^	40.3^a^	23.3^a^
42 days	47.4^e^	87.6	12.5^c^	5.59^b^	41.0^a^	23.7^a^
SEM	0.771	0.986	0.614	0.358	1.521	1.659
*p*-value^3^						
MOIS	<.001	0.027	<.001	0.159	0.173	0.006
TIME	<.001	0.155	<.001	<.001	<.001	0.003
MOIS*TIME	<.001	0.552	0.003	0.040	0.343	0.911
Linear	0.012	0.072	<.001	<.001	<.001	<.001
Quadratic	0.856	0.172	0.915	0.140	0.023	0.107

^a~e^Means in the same column with different superscripts differ significantly (p < 0.05).

^1^DM, dry matter; OM, organic matter; CP, crude protein; EE, ether extract; NDF, neutral detergent fiber; ADF, acid detergent fiber.

^2^MO25, fermented concentrate with moisture level at 25%; MO35, fermented concentrate with moisture level at 35%; MO45, fermented concentrate with moisture level at 45%.

^3^MOIS, effect of moisture level; TIME, effect of fermentation length, MOIS*TIME, interaction effect of moisture level and fermentation length; Linear, linear effect by fermentation length; Quadratic, quadratic effect by fermentation length. SEM, standard error of mean.

After fermentation, the concentrations of several composites, such as DM, CP, and ADF, were influenced (*p* < 0.005) by moisture level (Table 4). On average, during the fermentation period (Table 4), MO25 presented a greater DM concentration (*p* < 0.001; 73.8% *vs.* 62.8% *vs.* 51.9%) than MO35 and MO45, respectively. MO25 and MO35 also presented greater CP concentration (*p* < 0.01; 15.8% and 15.9% *vs.* 14.7%) and lower ADF concentration compared to MO45 (*p* = 0.006; 20.5% and 20.5% *vs.* 22.0%). The fermentation length affected the results of DM, CP, EE, NDF, and ADF. Concentrations of DM, CP, and EE dropped linearly (*p* < 0.05) with the extended fermentation length. On the other hand, the results of NDF and ADF were linearly increased by the extended fermentation length (*p* < 0.05).

**Table 5.** Effects of moisture levels, content and fermentation lengths on the fermentation characteristics of fermented concentrate using tamanu cake as a protein source.

Fermentation length	Fermentation characteristics					
	pH	Ammonia-N (%, DM)	Lactate (%, DM)	Acetate (%, DM)	Propionate (%, DM)	Butyrate (%, DM)
MO251						
2 days	5.88a	2.62c	1.20e	0.00e	ND	ND
7 days	5.84a	2.67c	1.32e	0.00e	ND	ND
14 days	5.77a	2.55c	1.95e	0.28d	ND	ND
21 days	5.70a	2.96c	2.75e	0.51c	ND	ND
42 days	5.21c	3.16bc	3.49d	0.73b	ND	ND
MO35						
2 days	5.65b	2.94c	3.80d	0.00e	ND	ND
7 days	5.51b	3.04c	3.92d	0.00e	ND	ND
14 days	5.02c	3.22bc	4.07cd	0.47c	ND	ND
21 days	4.62d	3.55ab	4.30c	0.86b	ND	ND
42 days	4.33e	4.02a	4.98b	1.00b	ND	ND
MO45						
2 days	5.54b	3.69ab	4.40c	0.20d	ND	ND
7 days	5.45b	4.04a	4.66bc	0.44c	ND	ND
14 days	4.88c	4.10a	5.12ab	0.47c	ND	ND
21 days	4.37e	4.10a	5.57ab	1.31ab	ND	ND

There was an interaction between moisture level and fermentation length on concentrations of DM, CP, and EE, showing that the decrease after 42 days of fermentation was higher in MO45. This result suggested that a combination of high moisture and a long fermentation period could significantly increase the potential for nutrient loss.

## Fermentation characteristics

In fermentation characteristics, pH and concentrations of ammonia-N and major organic acids (lactate and acetate) were influenced (*p* < 0.005) by moisture content before fermenting (Table 5). On average during the fermentation period, MO35 and MO45 generated a lower pH (*p* < 0.001; 5.03 and 4.88 *vs*. 5.68) and acetate (*p* < 0.001; 0.31 and 0.47 *vs*. 0.82) with a greater lactate concentration (*p* < 0.001; 4.21% and 5.17% *vs*. 2.15%) compared to MO25. The concentration of ammonia-N was greater consecutively in MO45, MO35, and MO25 (*p* < 0.001; 4.02% *vs*. 3.35% *vs.* 2.79%). The concentrations of minor organic acids, such as propionate and butyrate, were not found by measurement in all treatments. In general, pH conditions decreased linearly (*p* < 0.001) with longer fermentation length, while concentrations of ammonia-N, lactate, and acetate increased linearly (*p* < 0.005). There was an interaction between moisture level and fermentation length on pH (*p* < 0.001) and ammonia-N (*p* = 0.009), while the pH between MO35 and MO45 was not different after 42 days. The ammonia-N concentration of MO45 at 2 days was similar to MO25 at 42 days and MO35 at 21 days, showing that ammonia-N production massively occurred in high-moisture fermentation over a short period.

## Microbial counts

Microbial count for both LAB and yeast was affected (*p* < 0.005) by moisture content before ensiling (Table 6). On average, during fermentation, MO45 had the highest LAB count, followed by MO35 and MO25 (*p* < 0.001; 5.83 *vs.* 5.20 *vs.* 4.91 log10 cfu/gm). Yeast count was lower in MO25 and MO35 (*p* = 0.001; 4.87 and 4.68 log10 cfu/gm *vs.* 5.08 log10 cfu/gm), while bacillus count was higher in MO35 and MO45 (*p* < 0.001; 5.34 and 5.47 log10 cfu/gm *vs.* 4.93 log10 cfu/gm). The longer fermentation period accelerated linearly (*p* < 0.001) the growth of LAB and bacillus but showed a quadratic (*p* < 0.001) growth of yeast. The growth of yeast in MO35 and MO45 increased until 21 days and then decreased at 42 days of fermentation. The mold was not detected during fermentation, while the counts of *E. coli* and *Salmonella* were only observed on 2 days of MO45, which were 3.07 and 3.10 log10 cfu/gm, respectively. There was an interaction between moisture level and fermentation length on LAB (*p* = 0.018), yeast (*p* < 0.001), Bacillus (*p* < 0.001), *E. coli* (*p* < 0.001), and *Salmonella* (*p* < 0.001) count. LAB and yeast counts on MO35 and MO45 were found to be different on days 2 and 7, but similar after 14 days of fermentation. The findings showed that moisture content had a higher effect during the short fermentation period, as also reflected in the bacillus count. In this study, *E. coli* and *Salmonella* were only detected in early fermentation with a high moisture level.

**Table 6.** Effects of moisture levels, content, and fermentation lengths on microbial counts of fermented concentrate using tamanu cake as a protein source.

Fermentation length	Microbial counts (log10 cfu/gm)					
	Lactic acid bacteria	Yeast	Mold	Bacillus	*E. coli*	*Salmonella*
MO25^1^						
2 days	4.82^cd^	4.20^c^	ND	4.40^e^	ND	ND
7 days	4.48^cd^	4.59^bc^	ND	4.60^ef^	ND	ND
14 days	5.01^bcd^	5.10^bc^	ND	5.08^cd^	ND	ND
21 days	5.02^bcd^	5.32^ab^	ND	5.21^cd^	ND	ND
42 days	5.20^bc^	5.15^ab^	ND	5.30^cb^	ND	ND
MO35						
2 days	4.54^cd^	4.13^c^	ND	4.82^de^	ND	ND
7 days	4.32^d^	4.58^bc^	ND	5.17^cd^	ND	ND
14 days	5.77^ab^	5.03^ab^	ND	5.30^cb^	ND	ND
21 days	5.58^ab^	5.43^a^	ND	5.63^ab^	ND	ND
42 days	5.80^ab^	4.24^bc^	ND	5.76^a^	ND	ND
MO45						
2 days	5.51^ab^	4.76^bc^	ND	4.94^cde^	3.07	3.10
7 days	5.40^b^	5.00^ab^	ND	5.15^cd^	ND	ND
14 days	5.92^a^	5.73^a^	ND	5.76^a^	ND	ND
21 days	5.92^a^	5.73^a^	ND	5.76^a^	ND	ND
42 days	6.27^a^	4.16^c^	ND	5.77^a^	ND	ND
SEM	0.261	0.204	NA	0.129	0.024	0.037
*p*-value^2^						
MOIS	< 0.001	0.001	NA	< 0.001	< 0.001	< 0.001
TIME	< 0.001	< 0.001	NA	< 0.001	< 0.001	< 0.001
MOIS*TIME	0.018	< 0.001	NA	< 0.001	< 0.001	< 0.001
Linear	< 0.001	0.855	NA	< 0.001	0.006	0.007
Quadratic	0.065	< 0.001	NA	0.126	0.200	0.201

^a~f^Means in the same column with different superscripts differ significantly (*p* < 0.05).

^1^MO25, fermented concentrate with moisture level at 25%; MO35, fermented concentrate with moisture level at 35%; MO45, fermented concentrate with moisture level at 45%.

^2^MOIS, effect of moisture level; TIME, effect of fermentation length, MOIS*TIME, interaction effect of moisture level and fermentation length; Linear, linear effect by fermentation length; Quadratic, quadratic effect by fermentation length.

SEM, standard error of mean. cfu, colony forming unit.

## Aerobic stability

**Figure 1.**
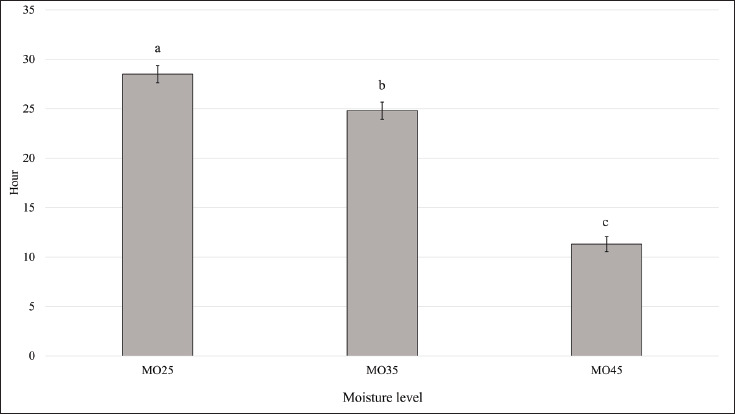
Effect of moisture levels on aerobic stability of fermented concentrate using tamanu cake as protein source. ^a,b,c^Means with different superscripts differ significantly (p < 0.05). MO25, fermented concentrate with moisture level at 25%; MO35, fermented concentrate with moisture level at 35%; MO45, fermented concentrate with moisture level at 45%.

Moisture content before fermenting was found to affect (*p* < 0.005) the concentrate aerobic stability after 42 days of fermentation (Fig. 1). Aerobic stability indicated the condition during the feed-out phase, and it ended if the temperature in the diet was 2°C higher than ambient. The highest aerobic stability was observed in MO25, followed by MO35 and MO45 (*p* < 0.001; 28.5 h *vs*. 24.8 h *vs*. 11.3 h), with MO25 and MO35 showing better aerobic stability, each exceeding 24 h.

## Ruminal digestibility

**Figure 2.**
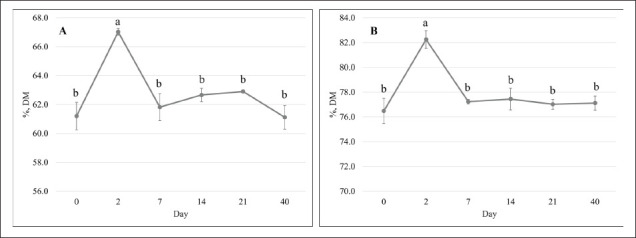
Effect of fermentation lengths on dry matter digestibility (A) and organic matter digestibility (B) of fermented concentrate using tamanu cake as protein source. ^a,b^Means with different superscripts differ significantly (p < 0.05).

This study found that MO35 showed fermentation quality similar to MO45 and good aerobic stability comparable to MO25. Consequently, MO35 was selected and continued for ruminal digestibility. IVDMD and IVOMD increased at 2 days of fermentation (Fig. 2), with both parameters showing similar values on 7, 14, 21, and 42 days as well as 0 days. In addition, longer fermentation length linearly dropped the production of total VFA (Table 7). Fermentation on 0 and 2 days generated a higher total VFA concentration compared to 21 and 42 days. Rumen pH, ammonia-N concentration, each VFA profile, and the ratio of acetate to propionate was not influenced by the fermentation length, but CH₄ emission decreased linearly as the fermentation length increased.

## Discussion

In general, the chemical compositions of TKC in the present study were in the normal range according to a previous study [19]. The TKC can be used as a source of protein in the dietary treatment. High concentrations of CP and EE were found in the fermented concentrate prepared during this study. As the main ingredient, TKC contributed 20.1% CP and 15.3% EE [19], which influenced the overall CP and EE levels in the ration.

**Table 7.** Effects of moisture levels, content, and fermentation lengths on ruminal fermentation and methane emission of fermented concentrate using tamanu cake as a protein source.

Item	Fermentation length, days						SEM	Contrast^1^
	0	2	7	14	21	42	L		Q
pH	6.71	6.69	6.72	6.70	6.70	6.74	0.032	0.700	0.977
Ammonia, mg/100 ml	20.5	20.2	19.6	19.6	18.8	18.5	1.436	0.099	0.772
Total VFA, mM/l	225.4^a^	268.3^a^	197.4^ab^	155.3^b^	139.4^b^	136.1^b^	26.25	0.001	0.097
Acetate, % of molar	35.3	35.8	33.0	34.4	31.7	34.0	3.066	0.131	0.945
Propionate, % of molar	31.3	33.9	35.2	33.3	36.2	34.5	2.345	0.024	0.544
Iso-butyrate, % of molar	4.36	3.49	4.22	4.72	4.91	4.53	0.997	0.141	0.950
Butyrate, % of molar	24.2	22.8	22.8	22.2	21.9	22.1	1.679	0.113	0.359
Iso-valerate, % of molar	4.81	4.00	4.66	5.31	5.21	4.80	0.868	0.312	0.799
Acetate: Propionate	1.07	1.07	0.95	1.03	0.88	0.99	0.166	0.100	0.986
Methane, ppm	7,2241.3	67,111.4	67,521.7	65,120.9	67,828.6	6,6056.6	4,953.1	0.460	0.197

^a,b^Means in the same row with different superscripts differ significantly (*p* < 0.05).

^1^L, linear effect by fermentation length; Q, quadratic effect by fermentation length.

SEM, standard error of mean.

VFA, total volatile fatty acid.

The present study indicated that higher moisture levels of fermented concentrate could diminish the concentrations of DM, CP, and EE. The concentrations of NDF and ADF were reported to increase following fermentation by higher moisture levels, which could potentially decrease palatability and digestibility. The proportion of NDF and ADF dramatically elevated in the fermented concentrate since the proportion of CP and EE decreased due to losses during fermentation, which was similar to previous studies [17,25,28]. In the industry, particularly in Indonesia, the production of fermented concentrate primarily uses a moisture level of 45% with a storage time of more than 3 weeks. For long-term storage, the reduction of moisture content can be an option to decrease nutrient loss. Therefore, the results obtained can be a recommendation to increase the quality of fermented concentrate. According to previous studies, moisture level and fermentation length directly affect the chemical composition of fermented feed, but the results can vary depending on the type of diet, anaerobic conditions, and microbial inoculation [8–10,15]. In general, average concentrations of OM, CP, EE, NDF, and ADF during fermentation on MO25 and MO35 were statistically similar, signifying that nutrient loss had less impact at low moisture levels under 35%.

Fermentation of more than 21 days was found to increase DM and CP losses across all moisture levels. DM loss on 42 days of fermentation was approximately 7.10%, 4.20%, and 1.8% for MO45, MO35, and MO25, respectively. CP loss on 42 days was approximately 3.6%, 1.70%, and 1.10% for MO45, MO35, and MO25, respectively.

In addition, EE loss for MO35 occurred at 42 days, and for MO45 it was observed after 21 days, showing that the moisture levels of fermented concentrate before ensiling had less impact on short-term fermentation. An increase in fermentation length showed similar effects as a higher moisture content on the chemical composition of the concentrate. Longer fermentation length decreased the concentrations of DM, CP, and EE, while it also increased the concentrations of NDF and ADF. These results occurred because both moisture content and fermentation time were key factors that had direct effects on the growth of microbes. Both bacteria and fungi use a nutrient in the diet for growth and metabolite production, but the growth of these microorganisms is affected by moisture levels [15]. Lower moisture content reduces microbial activities [29] and growth (Table 5), thereby decreasing the use of nutrients in the diet during fermentation. The growth of microbes could increase and stabilize over a longer fermentation time following the availability of substrate in the diet, then the microbes die off once the substrate is depleted [12,30].

In the fermentation characteristics, the pH of fermented feed was found to decrease with higher moisture levels and longer fermentation periods, which supported the production of lactate and acetate. These results related to the increase of LAB population by higher moisture level and longer fermentation length (Table 5), which was consistent with previous studies [12,31]. LAB converts water-soluble carbohydrates to produce organic acids, such as lactate and acetate [12,32], which are influenced directly by the growth of this bacterium (Table 3). Previous studies reported that higher moisture levels could increase organic acid production [8–10], probably due to the elevation of microbial activity [29].

Longer fermentation length could increase the production of organic acids related to log and lag phases during fermentation [11,30]. Similar to lactate and acetate, the concentration of ammonia was increased by higher moisture levels and longer fermentation length, which was supported by previous studies [8,9,11,30]. The increase in ammonia production occurred massively when the moisture level was at 45%, which showed protein loss. This observation was based on ammonia-N serving as a reflection of protein loss during fermentation [12,32]. In addition, propionate and butyrate were not detected in this study. The presence of butyrate signified the growth of clostridia that was avoided during the fermentation [12,32], showing that the fermentation was conducted in proper conditions. In general, average concentrations of lactate and acetate during fermentation on MO35 and MO45 were statistically similar. This implied that the minimum moisture level of 35% could help to optimize the production of organic acid.

In the microbial count, the growth rate of LAB is influenced by moisture, substrate, temperature, and anaerobic conditions [8–11]. Several previous reports were similar to this study, showing that the increase in moisture level could enhance the growth of LAB and Bacillus on fermented feed such as silage and fermented TMR [10,17,33]. According to a particular investigation, the minimum moisture content for optimizing LAB growth in rice grain silage was 27.5% [33], which was identical to the moisture content in MO25. This study discovered that the moisture level affected the growth rate of LAB and Bacillus in short-term fermentation. The results indicated that higher moisture levels could improve the growth rate of LAB in log and lag phases. In addition, yeast was detected in all treatments, and the population increased with higher moisture levels, which was similar to the previous report [17,33]. The growth of yeast tends to be facilitated by the use of a commercial inoculant containing *S. cerevisiae*, which plays a role as a fibrinolytic microbe to increase digestibility [34].

The growth of LAB increased with longer fermentation time in the present study, which was in agreement with several previous studies [11,30]. However, the growth of yeast decreased after long-term fermentation, probably due to inhibition caused by the low pH condition and acetate production. Acetate has antimicrobial effects that could inhibit the growth of fungi, including yeast and mold [35]. Mold, *E. coli*, and *Salmonella* are pathogenic microorganisms harmful to animals [36]. Contamination of these pathogenic microbes could come from the field, which was caused by poor management, aerobic exposure, or feed-out stages. A successful fermentation process led to a high production of organic acids capable of inhibiting the growth of these pathogenic microorganisms [36]. The fermentation in this study was conducted properly, as shown by the absence of mold in all treatments. *Escherichia* and *Salmonella* were not detected in MO25 and MO35 at any fermentation length, while MO45, fermented for 2 days, presented the growth of these two bacteria at low levels. High moisture could be a main factor in stimulating the growth of pathogenic microbes, such as *E. coli* and *Salmonella*. Fermentation of concentrate with high moisture content and a short-term period might still lead to a facultative condition for the growth of pathogenic microbes. However, the increase in organic acids by a longer fermentation length inhibited all pathogenic bacteria effectively.

Application of MO45 led to low aerobic stability less than 12 h after opening the silo, but this condition was not applicable in the field for local farmers. In this study, the application of MO25 and MO35 was found to generate better aerobic stability, which was more than 24 h. Lower moisture content could inhibit the growth of spoilage microorganisms during aerobic conditions. Previous studies also reported that higher moisture levels could decrease the aerobic stability of fermented feed [10,17,18]. High moisture levels could stimulate the growth of spoilage microbes during aerobic conditions, thereby decreasing the aerobic stability of fermented feed [12,37].

MO35 showed similar chemical compositions as well as aerobic stability to MO25, and fermentation characteristics were identical to MO45, leading to the recommendation of a moisture level of 35% for fermented concentrate. Consequently, MO35 was selected for further analysis of ruminal digestibility to evaluate the effects of fermentation length. During ruminal incubation, fermentation for 2 days was reported to increase IVDMD and IVOMD, which were supported by the lowest pH and the numerically highest VFA concentration. Digestibility in the rumen is known to have a positive relationship with total VFA production [18,38].

Concentrations of ADF on 2 days of fermentation numerically decreased (Table 4) compared to before fermentation (Table 3), and this change could be a reason for the improvement of ruminal digestibility. *Saccharomyces cerevisiae* as an inoculant could produce cellulolytic enzymes for degrading cellulose as a fraction of ADF in the diet [34,39]. However, fermentation of more than 3 days initiated lower pH conditions, which might cause the fibrinolytic enzymes not to properly degrade cellulose and hemicellulose. Cellulolytic enzymes work optimally in neutral pH conditions [38], while CH₄ emission was not affected by fermentation length. The results of acetate, propionate, and butyrate had no differences based on the length of fermentation. Changes in the proportion of acetate, propionate, and butyrate could affect the amount of CH₄ production related to metabolic hydrogen flow in rumen fermentation [38,40]. A limitation of the present study is that ruminal digestibility was carried out using the *in vitro* technique, where the result could not properly represent the real condition in animals. Therefore, the *in vivo* study of fermented concentrate using TKC is necessary to conduct further.

## Conclusion

In conclusion, this study identified that the application of a moisture level of 35% was recommended, particularly for long-term storage of fermented concentrate. MO35 could reduce nutrient loss and improve aerobic stability above 24 h, similar to MO25, while generating high fermentation quality identical to MO45. The short-term fermentation at 2 days improved ruminal digestibility related to the decrease of ADF concentrations by fibrinolytic inoculants. Additionally, short-term fermentation of less than 21 days was recommended to prevent nutrient loss, but could still improve the fermentation quality. However, in long-term fermentation, the improvement of ruminal digestibility was not observed due to the inactivation of fibrinolytic enzymes under acidic conditions.

## List of abbreviations

ADF, Acid detergent fiber

CA, Crude ash

cfu, Colony forming unit

CP, Crude protein

DM, Dry matter

EE, Ether extract

IVDMD, *In vitro* dry matter digestibility

IVOMD, *In vitro* organic matter digestibility

LAB, Lactic acid bacteria

ME, metabolizable energy

NDF, neutral detergent fiber

OM, organic matter

TKC, tamanu kernel cake

VFA, volatile fatty acid

WSC, water-soluble carbohydrate

h, hours

gm, grams

L, liter

kg, kilograms
